# Vitamin D deficiency during late pregnancy mediates placenta-associated complications

**DOI:** 10.1038/s41598-021-00250-5

**Published:** 2021-10-20

**Authors:** Tiphaine Raia-Barjat, Camille Sarkis, Florence Rancon, Lise Thibaudin, Jean-Christophe Gris, Nadia Alfaidy, Céline Chauleur

**Affiliations:** 1 Department of Gynaecology and Obstetrics, Hôpital Nord, University Hospital, Avenue Albert Raimond, 42270 Saint Etienne, France; 2 Jean Monet Saint-Etienne University, INSERM, SAINBIOSE (SAnte, INgénierie, BIOlogie, Saint- Etienne), U1059, Saint-Etienne, France; 3 Institut National de La Santé Et de La Recherche Médicale, U1292, Biosanté, Grenoble, France; 4 University Grenoble-Alpes, 38000 Grenoble, France; 5 INSERM, Centre d’Investigation Clinique 1408, Saint-Etienne, France; 6 Biochemistry Laboratory, University Hospital, Saint-Etienne, France; 7 Laboratory of Hematology, University Hospital, Nimes, France; 8 University of Montpellier, Montpellier, France; 9 I.M. Sechenov First Moscow State Medical University, Moscow, Russian Federation; 10 Institut Desbrest d’Epidémiologie et de Santé Publique UMR INSERM - Université de Montpellier, Montpellier, France; 11 Commissariat à l’Energie Atomique et aux Energies Alternatives (CEA), Biosciences and Biotechnology Institute of Grenoble, Grenoble, France; 12 Centre Hospitalo-Universitaire Grenoble Alpes, Service Obstétrique, CS 10217, 38043 Grenoble Cedex 9, France; 13 Université Grenoble Alpes, Grenoble, France

**Keywords:** Biomarkers, Predictive markers

## Abstract

During pregnancy, maternal vitamin D insufficiency could increase the risk of preeclampsia. Aim of the study was to evaluate the relationship between vitamin D status and the occurrence of placenta-mediated complications (PMCs) in a population at high risk. A prospective multicenter cohort study of 200 pregnant patients was conducted. The vitamin D level of patients with placenta-mediated complications was lower at 32 weeks compared to uncomplicated pregnancies (*P* = 0.001). At 32 weeks, the risk of occurrence of PMCs was five times higher in patients with vitamin D deficiency (RR: 5.14 95% CI (1.50–17.55)) compared to patients with normal vitamin D levels. There was a strong, inverse relationship between serum 25(OH)D levels at 32 weeks and the subsequent risk of PMCs (*P* = 0.001). At 32 weeks, the vitamin D level of patients with late-onset PMCs was lower than the one of patients with early-onset PMCs and of patients without PMCs (*P* < 0.0001). These results suggest a role of vitamin D in the maintenance of placental performance and therefore in the prevention of the onset of late PMC.

## Introduction

Placenta-mediated complications (PMCs) correspond to a heterogeneous group of multisystemic disorders. These can be maternal (pre-eclampsia (PE), eclampsia, placental abruption, hemolysis elevated liver enzymes low platelets (HELLP) syndrome) or foeto-placental (intrauterine growth retardation (IUGR), in utero fetal death, recurrent spontaneous miscarriages). They are frequent since PE complicates between 2 and 8% of pregnancies^1,2^. The prevalence of PE is evaluated ranging between 0.5 and 1.5% in France, between 4 and 18% in Africa depending on the ethnic group^3^. They are also serious diseases since they are largely responsible for maternal–fetal morbidity and mortality and are a major contributor to premature births^4^. PE is associated with premature delivery in 15 to 67% of cases, IUGR in 10 to 25% and neonatal mortality in 1 to 2% of cases^5^. Two forms of pre-eclampsia can be distinguished: the early form, or “early-onset”, secondary to a placentation anomaly with alteration of the angiogenic balance and the late form, or “late-onset”, with normal placentation but with an abnormality in the maintenance of placental functions on cardiovascular risk field^6,7^.

Vitamin D3 is a pre-hormone, a micronutrient that is also endogenously produced when skin is exposed to UV-B. It has initially been identified as playing a role in bone diseases and calcium metabolism. Vitamin D3 affects gene regulation after its conversion to 1,25-dihydroxyvitamin D3 (1,25(OH)D), which is the high affinity ligand of the nuclear transcription factor vitamin D receptor (VDR). Ligand-activated VDR binds to accessible genomic sites in the vicinity of its target genes and modulates their transcription, with possible multi-organ consequences. It is thus now accepted that vitamin D plays a role in many organs, especially in the placenta^8^, through the regulation of the expression of key associated-developmental genes^9–11^. The 1,25(OH)D concentrations in the maternal systemic circulation and the placenta increase during pregnancy. Vitamin D status is assessed using the serum circulating 25(OH)D levels, measured by a reliable assay. According to the Endocrine Society Clinical Practice Guideline, vitamin D insufficiency is defined as a 25(OH)D concentrations of 21–29 ng/ml (525–725 nmol/L) and vitamin D deficiency as a 25(OH)D below 20 ng/mL (50 nmol/L)^12^. There is no clear definition of vitamin D deficiency in pregnancy but a 25(OH)D concentration above 20 ng/mL is recommended by the Institute of Medicine (IOM) for pregnant women to prevent preterm birth^13,14^. In a large French cohort, 46.5% of pregnant women had a 25(OH)D below 20 ng/mL^15^. During pregnancy, maternal vitamin D insufficiency could increase the risk of preeclampsia, preterm birth^14^, small-for-gestational age (SGA) or intrauterine growth retardation (IUGR) and gestational diabetes mellitus^16^. Importantly, the patterns of dysregulated vitamin D appear different in early and late PE^17^. Only two studies focused on vitamin D profiles of women at high risk of Placenta-mediated complications^18,19^.

The main objective of this study was to evaluate the relationship between vitamin D status and the occurrence of PMCs. Secondary objectives were (1) to evaluate the relationship between vitamin D status and the occurrence of PE with or without IUGR; (2) to evaluate the relationship between vitamin D status and the occurrence of early and late PMCs.

## Methods

### Study population

Our study is an ad-hoc study of a previously collected cohort from the AngioPred study^20^. The AngioPred study is a prospective multicenter cohort study that we have conducted in the Obstetrics and Gynecology department of Saint Etienne and Nimes University Hospitals and the Laboratory of Hematology in Nimes University Hospital. Patients were enrolled between June 2008 and October 2010 Only patients that have been included in the University Hospital of Saint Etienne benefited from the vitamin D dosage, *i.e.* 182 patients out of the 200 patients constituting the initial cohort.

The patients included were all at high risk for occurrence or recurrence of PMCs. The risks included, diabetes, chronic hypertension, obesity, maternal age younger than 18 years or older than 38 years, chronic kidney disease, systemic lupus erythematosus, antiphospholipid syndrome, family history of cardiovascular disease or venous thromboembolism in first degree relatives, biological thrombophilia without any personal history of venous thromboembolism or of PMC, a history of one or more episodes of PMCs or personal history of venous thromboembolism. The exclusion criteria were the following, twin pregnancies, patients with a history of fetal death, IUGR which etiology was of chromosomal, genetic or infectious originor, and the presence of any PMC or venous thromboembolism at inclusion.

The study was approved in March 2008 by the Ethics Committee and Institutional Review Board of the University Hospital of Saint Etienne . The study is registered with the ClinicalTrials.gov (identifier NCT00695942). All clinical investigations were performed according to the Helsinki Declaration of 1996. All women were given their informed consent before participation. They all were included before 20 weeks and gave their written consent. Data on vitamin D supplementation during pregnancy were not available.

### Blood collection

Each patient underwent a blood sample in complement to the conventional laboratory test for pregnancy monitoring at 20, 24, 28, 32 and 36 weeks of gestation, totaling five samples per patient. The samples were immediately centrifuged, aliquoted and stored at − 80 °C.

### Biological analysis

Vitamin D analysis can only be performed on a blood sample taken on dry tubes; this limitation made it mandatory to exclude all patients enrolled at of Nimes center, as their samples have been collected in anticoagulated tubes. The assays were carried out by the biochemistry laboratory of Saint Etienne university hospital. 25(OH)D was quantified with the immunodiagnostic systems (IDS) automated competitive binding chemiluminescence 25-OHD method on the IDS-iSYS analyzer (IDS Ltd, Boldon, UK). A value of 7 ng/mL, corresponding to the limit of quantification that we determined in our laboratory was assigned to any undetectable concentration.

We have defined the vitamin D deficiency by a 25(OH)D level < 20 ng/ml and vitamin D insufficiency < 30 ng/ml.

### Evaluation criteria

The primary outcome was the occurrence or recurrence of any PMC diagnosed according to the following criteria: (1) PE with or without IUGR. PE was defined according to the ISSHP (International Society for the Study of Hypertension in Pregnancy criteria)^21^. PE was diagnosed if a previously normotensive woman had a new onset hypertension (> 140 mmHg systolic or > 90 mmHg diastolic) after 20 weeks gestation associated with proteinuria (spot urine protein/creatinine > 30 mg/mmol [0.3 mg/mg] or > 300 mg/day or at least 1 g/L [‘2 + ’] on dipstick testing) or other maternal organ dysfunction (renal insufficiency, liver involvement, neurological complications, hematological complications); (2) IUGR without PE defined by a birthweight ≤ to the 10^th^ centile (According to the AUDIPOG formula) with umbilical Doppler abnormalities. This formula calculates the exact percentile of birth weight from gestational age at birth, sex of newborn and birth weight^22^.

The secondary outcomes were: (1) the occurrence or recurrence of a PE with our without IUGR as defined just before; (2) the occurrence or recurrence of any early PMC defined as occurring before 34 weeks, and late PMCs defined as occurring at or after 34 weeks.

### Statistical analysis

Statistical analysis was performed using XLSTAT®. Qualitative data were described by absolute and relative frequencies (expressed in %). Quantitative variables were described by mean and standard deviation. The qualitative variables were compared by the Chi-square test or by Fisher's exact test if the numbers were insufficient. The quantitative variables were compared by the Student's t test or in the case of a non-normally distributed variable by the Wilcoxon-Mann–Whitney test. The normality of each variable was checked beforehand by a Shapiro–Wilk test. The averages of vitamin D dosages at each of the 5 visits (20, 24, 28, 32, 36 weeks) were compared between patients who presented a PMC and patients who did not present one. We also estimated the relative risks (risk ratio) and their 95% confidence intervals for the association between vitamin D level and the risk of PMC and with the risk of preeclampsia. The two pre-specified cutoffs representing low 25(OH)D concentration including < 20 and 20–29 ng/mL were used to split the data. The association between each cut-off and placenta-mediated complication was then examined using multivariable logistic regression analysis to adjust for potential confounding by relevant maternal characteristics. Only those variables with a crude association of *P* < 0.15 were included. The plasma vitamin D cutoff value for predicting PMC was determined at each gestational age using the ROC curve with calculation of area under the curve and 95% confidence intervals (95% CI %) (Receiver Operator Characteristics). The threshold chosen on the ROC curve is the threshold which represents the best compromise between sensitivity and specificity. The area under the ROC curve was compared to the area under the first diagonal which is 0.5 using a hypothesis test. Thresholds were determined at 32 and 36 weeks for predicting PMC ≥ 34 weeks. For comparison of vitamin D levels between patients with early-onset PMCs, late-onset PMCs and patients with no PMCs, group differences were assessed using Kruskal–Wallis test. As distribution was skewed, a Dunn post-hoc test was assessed for differences between two groups. Whatever the statistical analysis considered, the significance of the result was only accepted for a risk alpha less than 5%.

## Results

### Clinical characteristics

One hundred eighty two patients were included, allowing the analysis of 859 plasma samples. All samples were taken before the onset of PE or IUGR. Forty-three patients developed a PMC (23.6%). The demographics and inclusion criteria are summarized in Table 1. Almost two-thirds of our patients had a history of PMC.

**Table 1:** Patient characteristics at inclusion.

	Total N = 182	PMC N = 43	No PMC N = 139	*P*
Age (years)	32 ± 5.0	32 ± 5.3	32.1 ± 5.1	0.9
Parity	1.3 ± 0.9	1.2 ± 0.8	1.3 ± 1.0	0.73
BMI (Kg/m^2^)	25.3 ± 6.6	25.2 ± 7.5	25.4 ± 6.4	0.71
Smoking	22 (12.4)	4 (9.5)	18 (13.2)	0.52
Diabetes	6 (3.3)	3 (7)	3 (2.2)	0.13
Kidney disease	4 (2.2)	1 (2.3)	3 (2.2)	0.96
Hypertension	17 (9.4)	3 (7)	14 (10.2)	0.53
Lupus	12 (6.6)	5 (11.6)	7 (5.1)	0.14
Antiphospholipid syndrome	4 (2.2)	1 (2.3)	3 (2.2)	0.96
Personal history of VTE	35 (19.2)	10 (23.3)	25 (18.1)	0.46
Personal history of PMC	119 (65.4)	24 (55.8)	95 (69.3)	0.10
Family history of VTE or PMC	38 (21.1)	8 (18.6)	30 (21.9)	0.64

Categorical variables reported as frequency (percentage) and continuous variables reported as mean ± standard deviation.

*BMI* body mass index, *VTE* venous thromboembolism, *PMC* Placenta-mediated complication.

### Relationship between vitamin D status and the occurrence of placenta-mediated complications

**Figure 1:**
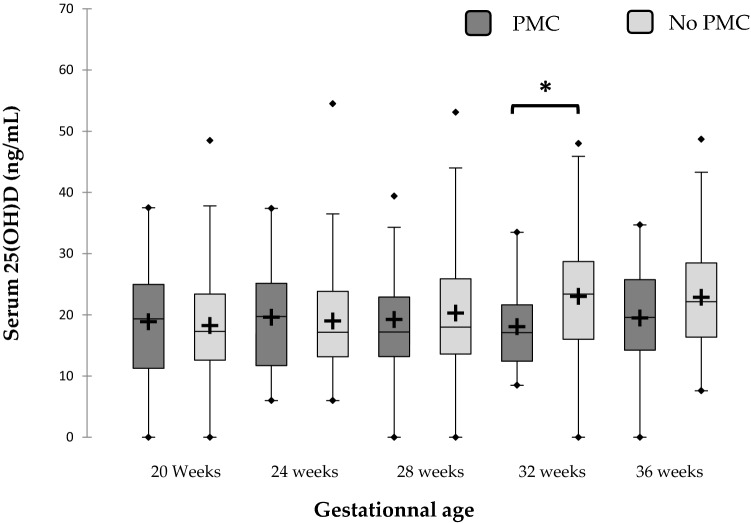
Evolution of the vitamin D profile during pregnancy depending on the occurrence of any PMC. The red crosses are the averages. The central horizontal bars are the medians. The lower and upper limits of the boxes are the first and third quartiles. The points are the minimum and maximum for each species. *PMC* placenta-mediated complication *P = 0.001.

Vitamin D levels were similar at 20, 24 and 28 weeks in patients with and without PMCs. The levels of vitamin D in patients with PMCs were lower at 32 weeks (*P* = 0.001) but would reach significance at 36 weeks (Fig. 1), if the sampling was strong at this gestational age. In fact at 36 weeks, the absence of significance was probably due to the lower number of patients, due to premature deliveries.

At 32 weeks, the average risk of occurrence of PMCs was five times higher in patients with a vitamin D deficiency compared with the one of patients with normal vitamin D levels (RR:5.14 95%CI (1.50–17.55)). After adjusting for lupus, diabetes and personal history of PMCs, the average risk of PMCs among women with 25(OH)D < 20 ng/mL was 15.45 higher than the risk of PMCs among women with 25(OH)D ≥ 30 ng/mL (RR: 14.45 95% CI (1.83–114.28)). At 36 weeks, the increase in risk was not significant (Table 2).

**Table 2:** Maternal vitamin D status at 32–36 weeks’ gestation and risk of placenta-mediated complications in women at high risk.

Serum 25(OH)D (ng/mL)	Number of patients	Number of PMC	Unadjusted risk ratio	Confidence interval 95%	Adjusted risk ratio^a^	Confidence interval 95%
**32 weeks**						
< 20	73	25	5.14	1.50–17.55	14.45	1.83–114.28
20–29	67	11	2.46	0.67–9.03	3.67	0.64–21.10
≥ 30	30	2	Reference		Reference	
**36 weeks**						
< 20	67	16	1.85	0.71–4.80	1.66	0.54–5.10
20–29	59	11	1.45	0.53–3.93	1.54	0.50–4.76
≥ 30	31	4	Reference		Reference	

*PMC* placenta-mediated complication.

^a^Multivariate analysis adjusted for lupus, diabetes and personal history of PMC, at each concentration of serum 25OHD.

**Figure 2:**
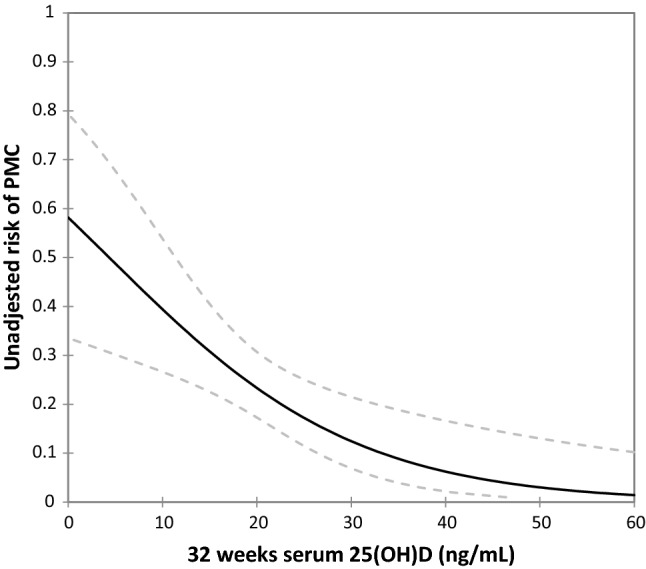
Dose–response association between maternal serum 25-hydroxyvitamin D concentration at 32 weeks and the risk of placenta mediated complications derived from a logistic regression model (*P* = 0.001). The solid line represents the point estimate, and the dotted lines represent the 95% confidence bands.

Indeed, as shown in Fig. 2, there was a strong, inverse relationship between serum 25(OH)D levels at 32 weeks and the subsequent risk of PMCs (*P* = 0.001).

**Figure 3:**
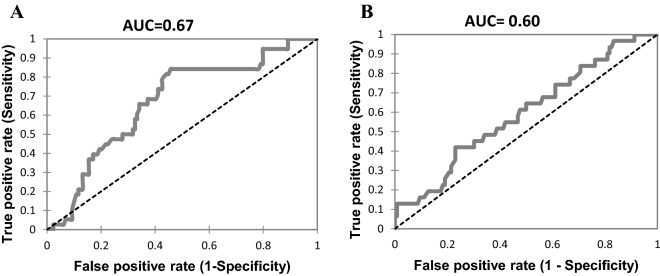
Vitamin D receiver operating characteristic (ROC) curves for placenta-mediated complication (**A**) at 32 weeks (**B**) at 36 weeks. *AUC* area under the receiver operating characteristic curve.

The ROC curves were used to calculate the threshold values having the best sensitivity and specificity for the prediction of PMCs at 32 and 36 weeks. The areas under the curve (AUC) was to 0.67 (95% CI (0.56–0.78)) at 32 weeks (*P* = 0.002) and 0.6 (0.47–0.72) at 36 weeks (*P* = 0.14). At 32 weeks, the threshold vitamin D value was 22.9 ng/mL with sensitivity of 84.2%, specificity of 54.3%, positive predictive value of 35.2% and negative predictive value of 92.1%. At 36 weeks, the threshold vitamin D value was 15.9 ng/mL with sensitivity of 41.9%, specificity of 77.0%, positive predictive value of 31.0% and negative predictive value of 84.3% (Fig. 3 a and b). Practically, vitamin D values tested at 32 weeks found higher than 22.9 ng/ml thus had the capacity to rule out the subsequent development of PMCs with an excellent discriminatory power.

### Relationship between vitamin D status and the occurrence of preeclampsia

At 36 weeks, among patients with very low vitamin D level (< 12 ng/ml), 22.2% (2/9) developed a PE but 0% (0/27) among patients with normal vitamin D (*P* = 0.02). At 32 weeks, relative risk values were not significantly modulated by vitamin D levels (Table 3). After adjusting for lupus, diabetes and personal history of PMC, at 32 and 36 weeks, the increase in risk was not significant.

**Table 3:** Maternal vitamin D status at 32–36 weeks’ gestation and risk of pre-eclampsia in women at high risk.

Serum 25(OH)D (ng/mL)	Number of patients	Number of pre-eclampsia	Unadjusted risk ratio	Confidence interval 95%	Adjusted risk ratio^a^	Confidence interval 95%
**32 weeks**						
< 20	61	13	2.88	0.81–10.27	4.41	0.78–25.06
20–29	60	4	0.90	0.21–3.95	1.28	0.21–7.90
≥ 30	27	2	Reference		Reference	
**36 weeks**						
< 20	62	8	3.61	0.68–19.35	1.45	0.41–5.17
20–29	52	4	1.67	0.24–10.74	1.20	0.31–4.59
≥ 30	28	1	Reference		Reference	

^a^Multivariate analysis adjusted for lupus, diabetes and personal history of PMC, at each concentration of serum 25OHD.

### Relationship between vitamin D status and the occurrence of early and late placenta-mediated complications

At 32 weeks, the vitamin D level of patients with late-onset PMCs was lower than the one of patients with early-onset PMCs and the one of patients without PMCs (*P* < 0.0001) (Table 4). There was no significant difference for the other pregnancy terms.

**Table 4:** Maternal Serum 25(OH)D (ng/mL) according to the term of PMC onset.

Gestational age	No PMC N = 139	PMC < 34 weeks N = 13	PMC ≥ 34 weeks N = 29	Kruskal–Wallis test *P* value	Dunn post-hoc analysis
20 weeks	18.43 ± 7.85	17.83 ± 7.10	20.17 ± 9.87	0.71	0.42^a^; 0.95^a^; 0.59
24 weeks	18.99 ± 8.3	18.78 ± 6.76	19.98 ± 9.34	0.91	0.66*; 0.88^b^; 0.89
28 weeks	20.38 ± 8.63	18.35 ± 5.53	19.89 ± 9.34	0.80	0.59^a^; 0.64^b^; 0.95
32 weeks	23.06 ± 8.72	24.76 ± 6.51	15.36 ± 4.61	< 0.0001	< 0.0001^a^; 0.37^b^; 0.001

Continuous variables reported as mean ± standard deviation. Comparison No PMC vs PMC > 34 weeks.

*PMC* placenta-mediated complication.

^a^No PMC vs PMC < 34 weeks.

^b^PMC < 34 weeks vs PMC > 34 weeks.

## Discussion

Serum vitamin D deficiency at 32 weeks was a strong, independent risk factor for PMCs during the observed pregnancies. Patients with a 25(OH)D deficiency have a five-fold higher risk of developing placenta-mediated complications and preferably late ones beyond 34 weeks. There was a dose–response relationship between maternal vitamin D concentrations at 32 weeks and the subsequent risk of developing any PMCs.

Most studies have described vitamin D profiles in the general population and not in populations at high risk. Several studies analysed the relationship between vitamin D deficiency in the first trimester of pregnancy and the subsequent occurrence of preeclampsia. Patients who developed preeclampsia had lower vitamin D levels at 14 weeks. The dose–effect relationship between the vitamin D concentration and the risk of pre-eclampsia (risk doubled) appeared for a threshold of 20 ng/mL. An increase in the concentration of 25(OH)D of at least 12 ng/mL, whatever the vitamin D status in the first trimester, was a protective factor against the occurrence of preeclampsia^23^. Women with vitamin D sufficiency during the third trimester and both in the first and 3rd trimesters had a significantly lower risk of preeclampsia^24^.

Others studies evaluated vitamin D status during the second trimester. Vitamin D deficiency before 22 weeks was a strong and independent risk factor for preeclampsia. A dose effect between the 25(OH)D concentration before 22 weeks and the risk of preeclampsia has been highlighted. After adjusting for confounding factors (ethnicity, season, BMI, social level, gestational age), a concentration of less than 20 ng/mL may double the risk of developing preeclampsia and a concentration below 15 ng/mL would be associated at a five-fold higher risk with preeclampsia. During childbirth, patients with preeclampsia had a serum level 15% lower than the control group. These results were found despite vitamin supplementation, three months before childbirth for 93% of the patients and during the periconceptional period for 46% of patients^25^. Another case–control study found a 25(OH)D concentration, in mid-pregnancy (between 18 and 20 weeks), lower in women who subsequently developed a severe pre-eclampsia. A concentration of less than 20 ng/mL was associated with a five-fold increase in the risk of severe pre-eclampsia compared to a concentration > 28 ng/mL^26^.

Recent study by Baca et al. investigated the relationship between maternal 25(OH)D concentration and the severity of preeclampsia. They demonstrated that when the level of 25(OH)D increased, the risk of preeclampsia decreased, with a threshold of 20 ng/mL. The adjusted risk of preeclampsia was 2.4 (95% CI 1.2–4.8), 1.1 (95% CI 0.69–1.7), and 1.3 (95% CI 0.89–1.8) respectively for 25(OH)D levels below 10 ng/mL, between 10 and 19.9 ng/mL and between 20 and 29.9 ng/mL compared to patients with levels above 30 ng/mL. Similar association was observed in relation to severe preeclampsia. In this study, the categorical analysis suggested that the impact of the decrease in vitamin D level was significant below 10 ng/mL compared to patients with levels ≥ 30 ng/mL^27^. Another study found that patients with levels of 20 ng/mL or more before 26 weeks had a 40% reduction in the risk of severe pre-eclampsia versus patients with levels below 20 ng/mL^28^. Other studies examined this link with the risk of developing severe preeclampsia^29,30^.

studies in high risk populations are very rare. High risk patients with 25(OH)D < 12 ng/mL had a 2.4-fold higher risk of early PE (< 35 weeks) compared with patients with 25(OH)D ≥ 30 ng/mL (adjustment of confounding factors: BMI and race). Importantly, women with previous preeclampsia had lower baseline 25(OH)D concentrations than women with chronic hypertension^19^. This study supports our results but, unlike our study, did not evaluate vitamin D status at the end of pregnancy. Another study did not find any significant difference in 25(OH)D concentrations between the group with or without preeclampsia despite a vitamin D deficiency present in 80% of patients^18^. In our study, we have highlighted the association between vitamin D deficiency and PMC. This association with the vitamin D status appears preponderant at the end of pregnancy (at 32 weeks and 36 weeks) in a high risk population. Our study also incorporated the risk of IUGR, while most studies did not. Thus, the vitamin D deficiency at 32 weeks is associated with the risk of PE but also of IUGR. The latest Cochrane meta-analysis highlights that maternal vitamin D insufficiency could increase the risk of preeclampsia^16^. We propose that vitamin D supplementation may reduce the risk of preeclampsia compared to no intervention or placebo and further emphasize that late supplementation could be beneficial.

Vitamin D during pregnancy plays three main roles. First, stimulation of calcium absorption ,a processes that is necessary for fetus’s bone mineral accrual during the last trimester of pregnancy. Second, vitamin D contributes to the tolerance of the fetus, being an allograft during pregnancy^31^. The third important role is its involvement in numerous transcriptional regulations^32^. Taking in mind that association is not causation, several plausible biological mechanisms might feed a role of vitamin D in preeclampsia (33). First, a placental implantation defect would lead to a decrease in placental perfusion. A weak placental perfusion would induce a production of materials which would be responsible for endothelial abnormalities^34,35^. Indeed endothelial functions are maintained via vitamin D by improving proliferation, migration and tubular formation^36^. The active form of vitamin D participates in the regulation of transcription and the function of genes involved in placental invasion and implantation, and in angiogenesis^37,38^. There is also evidence that vitamin D metabolites protect endothelial cells against oxydative stress and minimize the effects of exposure to factors related to preeclampsia^39,40^. Proteinuria in preeclampsia is thought to be modulated by vascular endothelial growth factor (VEGF). However, vitamin D would regulate the angiogenic process by acting directly on the transcription of the VEGF gene^41^. An inappropriate immune response between the mother and her fetus can mediate an implantation defect. The immunomodulatory functions of vitamin D thus appear relevant; the vitamin D deficiency could aggravate an already excessive inflammatory response^42,43^. Vitamin D supplementation improves the compliance, elasticity and thickness of the media and intima of vascular structures^44^. Finally, vitamin D deficiency could also be responsible for an increase in blood pressure by acting on the renin angiotensin aldosterone system^45^. Our study might suggest a role for vitamin D, particularly at the end of pregnancy, through the pathophysiological pathways described above. Vitamin D could especially regulate the maintenance of placental functions. As placental functions are thought to be impaired through a primary involvement of placental cells during early-onset PMCs, but through a secondary involvement due to peripheral blood and vascular cells dysfunctions in late-onset PMCS, vitamin D could mainly act through its systemic, not directly placental effects^45^.

Our study has some limitations. This is a multicenter study, but were only able to test vitamin D in patients from a single center. Also, the study did not examine all the determinants of vitamin D concentrations like seasonal dependence and there was no assay on the cord blood. We used the immunodiagnostic systems (IDS) automated competitive binding chemiluminescence 25-OHD method on the IDS-iSYS analyzer (IDS-iSYS) which is not the reference method for vitamin D assay^46^. In comparative study, the IDS-iSYS correlated well with both established methods (validated liquid chromatography-tandem mass spectrometry (LC–MS/MS) method and an IDS enzyme immunoassay (IDS-EIA) method)^47^. However, the strength of our study is the investigation of a high-risk pregnant population. It is a prospective study with repeated dosages throughout pregnancy. It is to date the only one study that considered the history of PMCs as a determinant of maternal vitamin D.

In conclusion, patients with vitamin D deficiency at 32 weeks have a higher risk of developing placenta-mediated complications. This deficiency preferentially favors late PMCs beyond 34 weeks. These results suggest an association of the vitamin D status with the maintenance of placental performance and therefore with the prevention of the onset of late PMC . To date, very few studies have evaluated vitamin D supplementation and its causation link with preeclampsia in high-risk populations, let alone the risk of IUGR. Many studies are underway which should clarify this hot topic in the near future.

## Data Availability

The datasets generated during and/or analyzed during the current study are available from the corresponding author on reasonable request.
